# Variation in RNA-Seq Transcriptome Profiles of Peripheral Whole Blood from Healthy Individuals with and without Globin Depletion

**DOI:** 10.1371/journal.pone.0091041

**Published:** 2014-03-07

**Authors:** Heesun Shin, Casey P. Shannon, Nick Fishbane, Jian Ruan, Mi Zhou, Robert Balshaw, Janet E. Wilson-McManus, Raymond T. Ng, Bruce M. McManus, Scott J. Tebbutt, for the PROOF Centre of Excellence Team

**Affiliations:** 1 NCE CECR PROOF Centre of Excellence, Vancouver, British Columbia, Canada; 2 UBC Department of Medicine (Division of Respiratory Medicine), University of British Columbia, Vancouver, British Columbia, Canada; 3 UBC Department of Pathology and Laboratory Medicine, University of British Columbia, Vancouver, British Columbia, Canada; 4 UBC Department of Computer Science, University of British Columbia, Vancouver, British Columbia, Canada; 5 UBC Department of Statistics, University of British Columbia, Vancouver, British Columbia, Canada; 6 UBC James Hogg Research Centre & Institute for HEART + LUNG Health, University of British Columbia, Vancouver, British Columbia, Canada; University of Southern California, United States of America

## Abstract

**Background:**

The molecular profile of circulating blood can reflect physiological and pathological events occurring in other tissues and organs of the body and delivers a comprehensive view of the status of the immune system. Blood has been useful in studying the pathobiology of many diseases. It is accessible and easily collected making it ideally suited to the development of diagnostic biomarker tests. The blood transcriptome has a high complement of globin RNA that could potentially saturate next-generation sequencing platforms, masking lower abundance transcripts. Methods to deplete globin mRNA are available, but their effect has not been comprehensively studied in peripheral whole blood RNA-Seq data. In this study we aimed to assess technical variability associated with globin depletion in addition to assessing general technical variability in RNA-Seq from whole blood derived samples.

**Results:**

We compared technical and biological replicates having undergone globin depletion or not and found that the experimental globin depletion protocol employed removed approximately 80% of globin transcripts, improved the correlation of technical replicates, allowed for reliable detection of thousands of additional transcripts and generally increased transcript abundance measures. Differential expression analysis revealed thousands of genes significantly up-regulated as a result of globin depletion. In addition, globin depletion resulted in the down-regulation of genes involved in both iron and zinc metal ion bonding.

**Conclusions:**

Globin depletion appears to meaningfully improve the quality of peripheral whole blood RNA-Seq data, and may improve our ability to detect true biological variation. Some concerns remain, however. Key amongst them the significant reduction in RNA yields following globin depletion. More generally, our investigation of technical and biological variation with and without globin depletion finds that high-throughput sequencing by RNA-Seq is highly reproducible within a large dynamic range of detection and provides an accurate estimation of RNA concentration in peripheral whole blood. High-throughput sequencing is thus a promising technology for whole blood transcriptomics and biomarker discovery.

## Introduction

Molecular profiles of circulating blood can be associated with physiological and pathological events occurring in other tissues and organs of the body [1], [2]. Peripheral whole blood is therefore a highly desirable tissue for developing diagnostic biomarker tests, due to its ease of accessibility and the low risk associated with its collection, as compared to invasive organ biopsies. To identify transcripts in peripheral blood that can potentially be used as diagnostic biomarkers, it is advantageous to utilize a technology that is highly sensitive and provides accurate quantification of RNA species. Conventional microarray technologies have been widely used for such purposes [3]–[6]. High-throughput DNA sequencing is a promising alternative transcriptome profiling technology that provides the greater sensitivity, transcript coverage and range, and data quality required for such investigations [7], [8]. In addition, such data may generate a more complete and comprehensive understanding of changes in transcript populations present in peripheral whole blood that are associated with disease, potentially providing insight into the molecular processes involved.

Globin dominates the peripheral whole blood transcriptome, accounting for 80-90% of transcript species. This overabundance may affect our ability to accurately detect other transcripts, particularly those with lower expression. This concern is not new. Experimental methods to specifically deplete globin RNA (globin depletion; GD) have been proposed as a possible solution and assessed on various technology platforms, including microarrays [9]–[12] and deep Serial Analysis of Gene Expression (SAGE) [7], but how it may affect RNA-Seq has not previously been characterized. This is of particular interest with the rapid adoption of RNA-Seq technology and the popularity of blood as a tissue for investigation. Arguably, globin transcript abundance is of particular concern in high-throughput sequencing applications, which rely on random sampling of the entire transcript pool to assess relative expression.

Chemical processing of delicate and often limited mRNA samples can potentially introduce variability, skewing data acquisition and preventing an accurate and consistent assessment of the data. The minimization of sample variation is of particular concern when attempting to identify and validate potential biomarkers for specific clinical purposes. In this study we investigate the applicability of RNA-Seq for transcriptome analysis from whole blood samples. Using a widely available globin depletion method we ask if globin depletion can reveal low-abundance transcripts otherwise masked by globin transcripts and we assess technical and biological variability associated with using globin depletion. We investigate the level of technical variability inherent in RNA-Seq data production and biological variability across transcriptome samples donated by six healthy individuals. Finally, we perform a limited differential gene expression analysis between globin depleted (GD) and non-globin depleted (NGD) samples in order to study any systematic effects of globin depletion on gene expression.

## Materials and Methods

### Ethics Statement

This study was conducted at the University of British Columbia and was approved by the UBC- Providence Health Care Research Ethics Board. Research participants gave informed written consent.

### Study design

We wished to investigate the effects of globin depletion on RNA-Seq peripheral whole blood transcriptome data. To do so we designed a study of a small collection of biological replicates (6 healthy individuals; 3 males, 3 females), as well as 6 technical replicates created from pooled total RNA extracted from peripheral whole blood across all 6 biological samples. These samples were then separated into two aliquots, submitted to globin depletion or not. A set of synthetic RNA spike-in controls (External RNA Controls Consortium [ERCC] spike-in mix) were additionally added to each of the 12 samples prior to sequencing, in order to assess detection sensitivity threshold under NGD vs. GD treatment. Finally, all 24 RNA samples were sequenced twice across two lanes, providing us with a final set of most similar technical replicates (12 lane technical replicate pairs).

**Figure 1:**
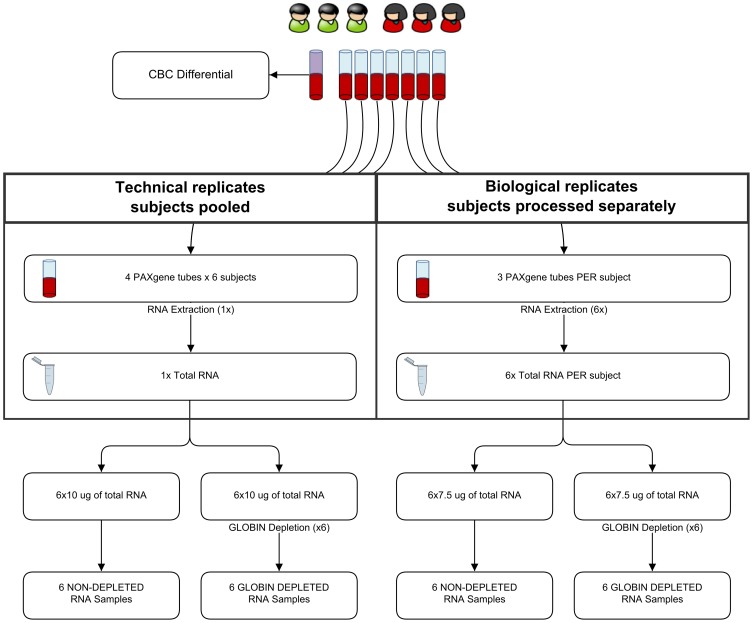
Experimental design.

Comparison of the technical replicates under different treatments, either globin depleted (GD) or not (NGD), will allow us to assess technical variability associated with globin depletion and study differences in transcriptome profiles. We hypothesized that depletion of the overabundant globin mRNAs would free up sequencing space for deeper coverage of the remaining transcript population at a given depth of coverage. Comparison of the biological replicates will allow us to examine the effect of GD on biological variability across the 6 individuals. Finally we performed differential gene expression (DGE) analysis between GD and NGD samples, in order to study any systematic effects of globin depletion on gene expression. A schematic of the study is shown in **Figure 1** and the technical details of blood collection, RNA extraction, ERCC spike-in and sequencing are detailed below.

### Blood sample collection and RNA extraction

Peripheral venous blood samples for 6 healthy volunteers (3 male and 3 female) were collected into PAXgene Blood RNA tubes (PreAnalytiX GmbH– BD Biosciences, Mississauga, ON, Canada) and BD vacutainer plastic EDTA tubes (BD Biosciences). Each subject donated 7 PAXgene blood samples and 1 EDTA blood sample. An aliquot of the EDTA collected blood was processed for total and differential cell counts on a Cell-Dyn automated hematology analyzer (Abbott, IL, USA). For each subject, 3 of the 7 PAXgene tubes were pooled. Total RNA was isolated from these subject-specific pooled samples, resulting in 6 subject-specific total RNA samples. The remaining 4 tubes were pooled across all subjects and total RNA isolated from this single pooled sample to create a single pooled total RNA sample, which was then aliquoted out to create 6 technical replicate total RNA samples. Total RNA, including small RNAs, was purified from PAXgene collected blood using the PAXgene Blood miRNA Kit (cat#763134, Qiagen, Chatsworth, CA, USA) according to manufacturer's protocols. The quality and yield of the isolated RNA was assessed using a NanoDrop8000 Spectrophotometer (Thermo Scientific, Wilmington, DE, USA), and Agilent 2100 Bioanalyzer (Agilent Technologies, Santa Clara, CA, USA). The 12 RNA samples (6 biological replicates and 6 technical replicates) were each further divided into two and either submitted to globin transcript depletion or not.

### ERCC RNA spike-in control mix addition, RNA concentration and globin mRNA depletion

In order to assess the technology platform dynamic range and lower limit of detection, External RNA Controls Consortium (ERCC) RNA spike-in control mix (cat#4456740, Invitrogen, San Diego, CA, USA) was added to each RNA sample before further processing. The 12 total RNA aliquots (6 biological replicates, 6 technical replicates) destined for globin transcript depletion were further processed as follows. RNA was concentrated using RNeasy MinElute Cleanup Kit (cat#74204, Qiagen) to reduce sample volume before globin transcript depletion. Human alpha and beta globin mRNA was then depleted from total RNA using the GLOBINclear kit (cat#AM1980, Ambion- Applied Biosystems, Mississauga, ON, Canada) and following the manufacturer's protocols. Prior to sequencing, the amount and quality of all 24 RNA samples were assessed using a NanoDrop8000 Spectrophotometer and Agilent 2100 Bioanalyzer.

### cDNA library construction and sequencing

1.75–3 µg total RNA samples were arrayed into a 96-well plate and PolyA+ RNA was purified using the 96-well MultiMACS mRNA isolation kit on the MultiMACS 96 separator (Miltenyi Biotec, Germany) with on column DNaseI-treatment as per the manufacturer's instructions. The eluted PolyA+ RNA was ethanol precipitated and resuspended in 10 µL of DEPC treated water with 1∶20 SuperaseIN (Life Technologies, USA). Double-stranded cDNA was synthesized from the purified polyA+ RNA using the Superscript Double-Stranded cDNA Synthesis kit (Life Technologies, USA) and random hexamer primers at a concentration of 5 µM. The cDNA was quantified in a 96-well format using PicoGreen (Life Technologies, USA) and VICTOR^3^V Spectrophotometer (PerkinElmer, Inc. USA). The quality was checked on a random sampling on the Agilent Bioanalyzer using the High Sensitivity DNA chip Assay. cDNA was fragmented by Covaris E210 (Covaris, USA) for 55 seconds, a “Duty cycle” of 20% and “Intensity” of 5. Plate-based libraries were prepared following the BC Cancer Agency, Genome Sciences Centre paired-end (PE) protocol on a Biomek FX robot (Beckman-Coulter, USA). Briefly, the cDNA was purified in 96-well format using Ampure XP SPRI beads, and was subject to end-repair and phosphorylation by T4 DNA polymerase, Klenow DNA Polymerase, and T4 polynucleotide kinase, respectively, in a single reaction, followed by cleanup using Ampure XP SPRI beads and 3′ A-tailing by Klenow fragment (3′ to 5′ exo minus). After cleanup using Ampure XP SPRI beads, picogreen quantification was performed to determine the amount of Illumina PE adapters used in the next step of adapter ligation reaction. The adapter-ligated products were purified using Ampure XP SPRI beads, then PCR-amplified with Phusion DNA Polymerase (Thermo Fisher Scientific Inc. USA) using Illumina's PE primer set, with cycle condition 98°C 30 sec followed by 10 cycles of 98°C 10 sec, 65°C 30 sec and 72°C 30 sec, and then 72°C 5 min. The PCR products were purified using Ampure XP SPRI beads, and checked with Caliper LabChip GX for DNA samples using the High Sensitivity Assay (PerkinElmer, Inc. USA). PCR products were normalized and pooled into two pools of twelve samples each. Pooled libraries of desired size range were purified using 8% Novex TBE Gels (Life Technologies, USA), and the DNA quality was assessed and quantified using an Agilent DNA 1000 series II assay and Quant-iT dsDNA HS Assay Kit using a Qubit fluorometer (Invitrogen). Libraries were then diluted to 8 nM. The final concentration was verified by Quant-iT dsDNA HS Assay. 75 base paired end reads were generated on Illumina HiSeq 2000 instruments using Version 3 flowcells, cluster generation and SBS kits and utilizing HCS V1.4.8 software.

### Summary of sequencing data and differential gene expression analysis

Twelve samples were sequenced for each treatment (NGD and GD; 6 technical replicates and 6 biological replicates) on 2 sequencing lanes per group, multiplexing the 12 samples on each lane for a balanced block design [13]. We therefore generated sequence data using 4 sequencing lanes in total, and each sample was sequenced on 2 separate lanes (at different times), serving as an additional (most similar) technical replicate for each sample. Sequence data quality check was performed using fastqc (v0.10.1). Sequence alignment to the UCSC reference genome (hg18) was carried out using Tophat [14] (v1.3.2). Assembly of transcripts and estimation of their abundance was performed using either Cufflinks [15] (v2.0.1) or the “summarizeOverlaps” function from the “GenomicRanges” package [16] (v1.12.5) for the R Statistical Programming Language [17] (v3.0.2). Cufflinks normalizes read counts, providing an estimate of the relative abundance of each transcript in each sample that allows for comparisons of relative abundance across transcripts. This normalization uses the total number of sequence reads, as well as transcript length, and is denoted as FPKM (**f**ragments **p**er **k**ilobase of transcript per **m**illion mapped reads). We used a stringent cutoff of FPKM≥1 to define transcripts that are robustly expressed [18]. Cuffmerge was used to merge separate lanes into one combined lane mapping result. Sequence data manipulation was performed using samtools [19] (v0.1.18). Sequence mapping to the genome and to the transcriptome was visualized in IGV [20] (v2.2). Finally, differential gene expression analysis was performed using the edgeR package [21] (v3.2.4). We limited our analysis to 7397 well annotated genes: 66803 UCSC transcripts were reduced to 13856 well annotated transcripts and collapsed to 7397 unique gene symbols. Genes with multiple mapping transcripts were assigned the maximum read count across all mapping transcripts. Plots were generated using the excellent ggplot2 package [22] (v0.9.3.1). The raw reads and summarized FPKMs for all samples are available on GEO (GSE53655).

## Results

### Effect of globin depletion on RNA-Seq data quality

In order to assess the effect of globin depletion on RNA-Seq data quality, the mean amount of RNA extracted (ng) from a fixed-volume (2.5 mL) PAXgene tube, UV 260/280 and UV 260/230 absorbance ratios, RNA integrity number (RIN), number of mapped reads, and number of robustly detectable transcripts (FPKM≥1) were compared between GD and NGD samples, in both technical and biological replicates (summarized in **Table 1**; detail available in **Table S1** and **Table S2**, respectively). Background could not be directly estimated as in [18], so a conservative threshold of 1 fragment per kilobase of transcript per million fragments mapped (FPKM) was selected. Transcripts with FPKM≥1 were deemed detectable with high confidence. At a given depth of coverage, we expected that depleting globin transcripts would allow for increased sampling of lower expressing transcripts, resulting in increased detectable transcript counts in GD samples.

**Table 1:** Effect of globin depletion on RNA-Seq data quality.

		Non-globin depleted	Globin depleted	Effect of globin depletion	Statistical significance (p≤0.05*; p≤0.01**)
**Technical replicates**	**RNA Extraction Yield (ng)**	10245	6031	↓	**
	**Nanodrop: UV 260/280**	2.08	2.05	↓	**
	**Nanodrop: UV 260/230**	1.37	0.88	↓	**
	**RIN**	8.75	8.14	↓	*
	**Total Reads**	28600000	29700000	-	-
	**Mapped Reads**	4240000	5040000	-	-
	**% Mapped**	14.81	14.34	-	-
	**Transcript Count (FPKM≥1)**	12117	16788	↑	*
	**Transcript Count (FPKM<1)**	35560	33315	↓	*
**Biological replicates**	**RNA Extraction Yield (ng)**	7409	3834	↓	**
	**Nanodrop: UV 260/280**	2.1	2.02	↓	**
	**Nanodrop: UV 260/230**	1.4	0.73	↓	**
	**RIN**	8.78	8.47	↓	**
	**Total Reads**	51900000	46500000	-	-
	**Mapped Reads**	8200000	7960000	-	-
	**% Mapped**	15.88	16.24	-	-
	**Transcript Count (FPKM≥1)**	13187	17470	↑	**
	**Transcript Count (FPKM<1)**	36713	32255	↓	**
	**% Globin Reads**	0.81	0.17	↓	**

The amount of extracted RNA, 260/280 and 260/230 UV absorption ratios, and RNA integrity number (RIN) were all significantly lower in globin-depleted samples, in both biological replicates and pooled technical replicates. However, the RNA amounts were above the recommended lower limit for library construction, 260/280 UV absorption ratio were greater than 2.0 and RIN above 8 across all GD samples. While the 260/230 UV absorption ratios were low (<1.0) in GD samples, this had no effect on either the total number of reads, total number of mapped reads or percent of mapped reads. The total number of mapped reads varied significantly across samples (coefficient of variation [CV] across technical replicates was 0.18 in NGD samples and 0.58 in GD samples; significant; p≤0.05), but not between lanes (**Figure S1**). We observed that the total number of reads and number of mapped reads was generally higher in the biological replicates when compared to the pooled technical replicates, and this was especially pronounced in NGD samples. There was no statistically significant difference in the total number of reads and total number of mapped reads between GD samples and NGD samples, in both technical and biological replicates. We noted that sequence lanes 1 (GD) and 2 (NGD), which were on the same flowcell, consistently yielded more mapped reads when compared to lanes 4 (GD) and 5 (NGD), which were on a different flowcell and sequenced several weeks later. On average, approximately 0.01% of reads were filtered out prior to mapping due to poor quality across all samples.

Finally, the total transcript counts were normalized across all samples and both marginal and detectable transcript counts computed for NGD and GD samples. The number of detectable transcripts (FPKM> = 1) in each lane varied from 13,823 to 18,797 (mean  = 16,806) across all GD samples and from 11,638 to 14,125 (mean  = 12,827) across all NGD samples. This translates to approximately 20–25% of transcripts (*i.e.*, 30–40% of genes) being detectable (the University of California, Santa Cruz [UCSC] hg18 reference genome lists 66,800 transcripts), and approximately 25% additional detectable transcripts in GD samples compared to NGD samples. In both technical and biological replicates, globin depletion resulted in a statistically significant increase in the number of detectable transcripts.

**Figure 2:**
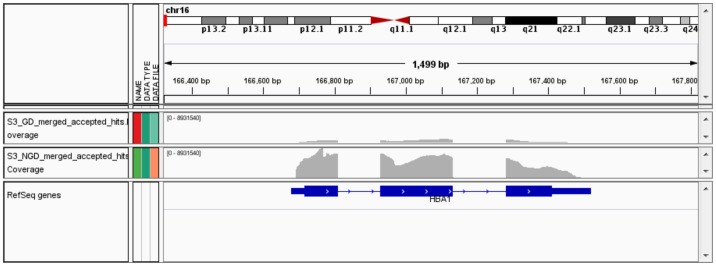
Globin depletion reduces the preponderance of reads mapping to globin transcript. The RNA-Seq reads mapping to one of the globin gene transcripts, HBA1, are visualized in the Integrated Genome Viewer software for an exemplar RNA sample that was globin depleted (top) or not (middle). The gene model is shown at the bottom.

The efficiency of globin depletion in reducing the relative abundance of globin transcripts in total RNA samples was less than reported by the provided kit's manufacturer. The proportion of total reads mapping to globin transcripts (HBA1, HBA2, HBB) was determined for each of the biological replicates and compared in globin depleted (GD) and non-globin depleted (NGD) samples. Globin depletion reduced the proportion of globin-mapped reads (mean 81% vs. 17% of reads mapping to globin transcripts; *p*<<0.01). A visualization of the effect of globin depletion on HBA1 transcript coverage in an exemplar subject is shown in **Figure 2**.

### Globin depletion does not introduce any significant bias in technical replicates

**Figure 3:**
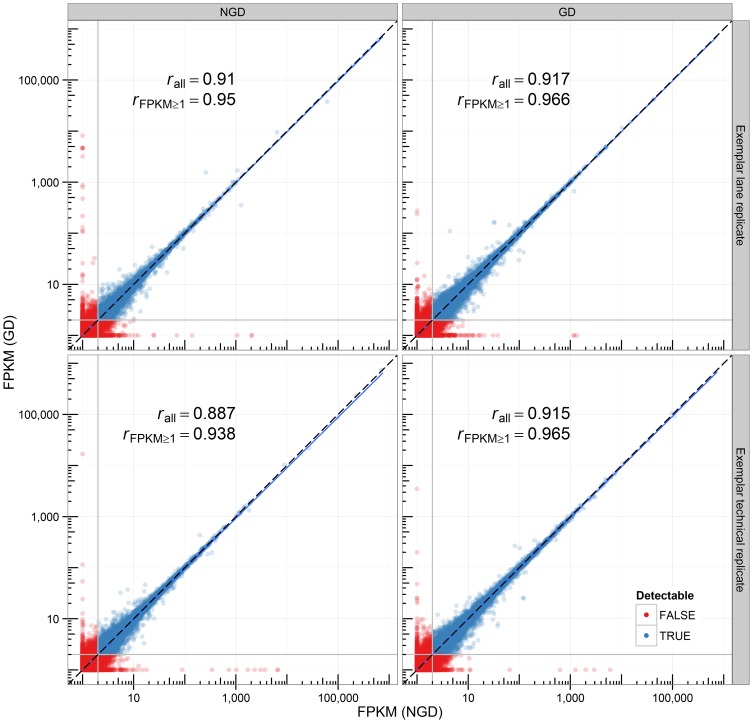
Globin depletion has no impact on the correlation of technical replicates. Correlation plot of the transcript FPKMs of lane technical replicate (top) or pooled technical replicate (bottom) exemplar pairs. Data is shown on a log scale. Spearman's *rho* is reported for all (*r*_all_) or reliably detectable transcripts (*r*_FPKM≥1_) only.

Next, we wished to investigate whether experimental globin depletion introduced any bias in RNA-Seq data. In order to address this question, we analyzed the correlation of pairs of replicates that were either globin-depleted or not. Recall that all RNA samples (technical and biological, NGD and GD) were sequenced in 2 lanes. The transcript abundances between these lane replicate pairs should exhibit the highest concordance and are used as a benchmark. The count data was log-transformed and the Spearman correlation of NGD or GD lane replicate and technical replicate pairs computed. Correlations were excellent across both lane and technical replicate pairs (Spearman's rho  = 0.871–0.910 across all transcripts; 0.921–0.950 when only considering transcripts with FPKM≥1; Pearson's product moment correlation  = 0.992–0.999; **Figure 3**). Globin depletion significantly improved the correlation in both lane and technical replicate pairs (*p*<<0.01). As expected, correlation was worse in NGD technical replicates compared to lane replicates (*p*<<1e-5). Interestingly, this was not the case when comparing GD technical replicate pairs to GD lane replicates (*p* = 0.397). Finally, we noted that there appeared to be greater transcript density in the 2.5–1000 FPKM range in GD samples. This is consistent with our hypothesis that globin depletion might free up sequencing space for deeper coverage of the non-globin transcript population.

### Globin depletion yields 3500 additional robustly detectable transcripts from a single representative peripheral whole blood RNA-Seq experiment

**Figure 4:**
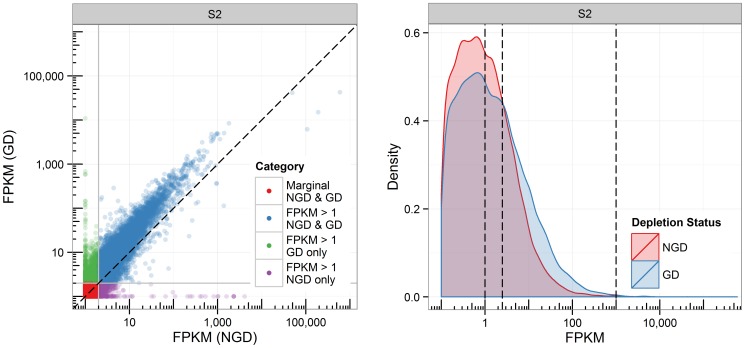
Globin depletion yields 3500 additional robustly detectable transcripts from a single representative peripheral whole blood RNA-Seq experiment. (A) Correlation plot of the transcript FPKMs of an exemplar biological sample, either globin depleted (y-axis) or not (x-axis). (B) Distribution of transcript FPKMs in the same exemplar biological sample, either globin depleted (blue) or not (red). Data is shown on a log scale. Significant FPKM cutoffs of 1, 2.5 and 1000 are marked by dashed line.

We next elected to investigate the effect of globin depletion on the FPKM values of individual transcripts, as well as the overall distribution of sample transcript FPKMs. We visualized the effect of globin depletion on transcript FPKMs by plotting NGD vs. GD (an exemplar sample is shown in **Figure 4A**). The measured expression of transcripts was generally higher in globin depleted samples (most transcripts were above the diagonal). Approximately 3500 additional transcripts were detectable at FPKM≥1 in the exemplar sample following globin depletion (*i.e.*, FPKM<1 in NGD, but ≥1 in GD). They were mostly non-coding, with low and highly variable expression (*i.e.*, CV>1; FPKM 1–1000). In addition, approximately 650 transcripts (mostly non-coding) were no longer detectable in GD samples.

**Figure 5:**
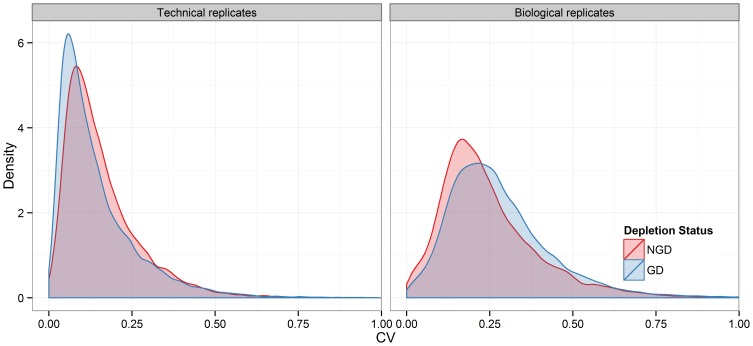
Globin depletion reduces technical variability while increasing measurable biological variability. Distribution of transcript coefficient of variation (CV) across pooled technical replicates (left) and biological replicates (right), either globin depleted (blue) or not (red).

The distribution of transcript FPKMs (**Figure 4B**) illustrates the additional discovery space that globin depletion in peripheral whole blood RNA-Seq data provides: globin depletion resulted in a marked shift in the transcript FPKM density distribution curve, with fewer transcripts in the 0–2.5 FPKM range and more transcripts between 2.5–1000 FPKM. This is consistent with the statistically significant increase in the number of detectable transcripts (and accompanied decrease in the number of marginal transcripts) that was observed in both technical and biological replicates (**Table 1**). Finally, variation of transcript expression across technical replicates was generally low, but coefficient of variation was significantly lower in GD technical replicates (median CV_technical replicates_ = 0.13 in NGD samples, median CV_technical replicates_ = 0.11 in GD samples; *p*<<0.01; **Figure 5**). As expected, biological replicates CVs were generally higher than that seen in technical replicates. Interestingly, CVs were higher in GD biological replicates (median CV_biological replicates_ = .21 in NGD samples, median CV_biological replicates_ = 0.25 in GD samples; *p*<<0.01).

### ERCC spike-in controls to measure accuracy, sensitivity, and reproducibility of whole blood RNA-Seq

We used a panel of external RNAs, developed by the External RNA Controls Consortium (ERCC) [23], [24], as spike-in controls. These consisted of a set of 92 unlabeled, polyadenylated transcripts added to each sample after sample isolation and prior to globin depletion. The dynamic range of our data was approximately FPKM 1.5–590000, reliably detecting approximately 96% of the synthetic ERCC RNAs. Concentration of the 92 ERCC synthetic RNAs ranged from 0.014–30000 attomoles/µL. ERCC spike-in controls indicated good correlation between the known concentrations and sequencing coverage of the synthetic RNAs overall (*r* = 0.924–0.981; **Figure S2**).

### Differential gene expression as a result of globin depletion

**Figure 6:**
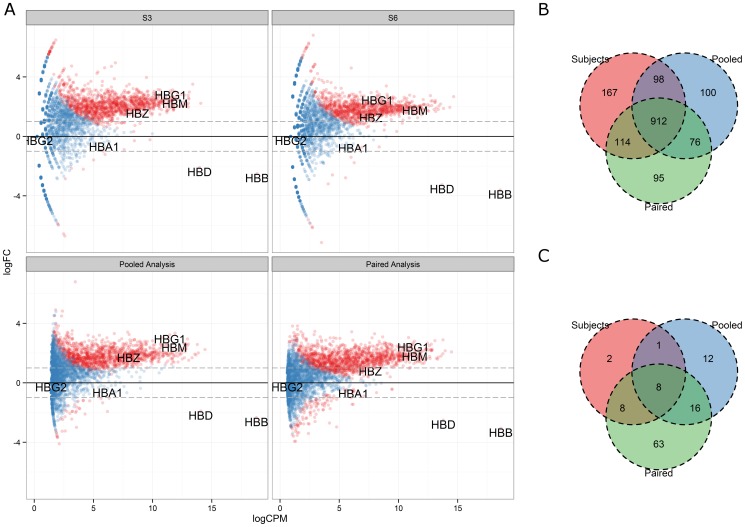
Globin depletion results in increased differential gene discovery. (A) The results of differential gene expression analysis comparing NGD and GD samples are visualized for various experimental designs. The log_2_ fold-change of genes between NGD and GD is plotted against mean log_2_ counts per million mapped reads (CPM; a library-size normalized expression measure) across samples and significant genes (FDR≤0.01) are shown in red. Overlaps in significantly differentially expressed genes across the various designs are visualized in Venn diagrams for (B) up-regulated and (C) down-regulated genes.

Finally, we carried out a differential gene expression analysis comparing NGD and GD samples. We used a number of different experimental designs to investigate the effect of globin depletion on gene expression as assessed by RNA-Seq. We first compared individual NGD vs. GD subjects, using different lanes as replicates (**Figure 6A:** **Subject 3 & Subject 6**). We also compared pooled technical replicates NGD vs. GD samples, merging reads from replicate lanes (**Figure 6A:** **Pooled Analysis**). Finally, we studied the effect of globin depletion across all subjects, using merged lane reads and subtracting their baseline differences (**Figure 6A:** **Paired Analysis**). As expected, the differentially expressed genes identified in these various designs heavily overlapped. Over a thousand genes were consistently up-regulated as a result of globin depletion (FDR≤0.01; **Figure 6B**), while only a handful of genes were consistently down-regulated across various experimental designs (**Figure 6C**). These down-regulated genes are tabulated in **Table 2** and summarized to enriched GO molecular function terms in **Table 3**.

**Table 2:** Transcripts down-regulated as a result of globin depletion.

UCSC Transcript	Entrez	Ensemble	Gene Symbol	Mean logFC	Mean logCPM	PValue	FDR
**uc001mae.1**	3043	ENSG00000244734	HBB	−2.88	18.71	0.00E+00	0.00E+00
**uc001maf.1**	3045	ENSG00000223609	HBD	−2.44	13.88	8.50E-130	4.00E-128
**uc003spa.1**	54476	ENSG00000011275	RNF216	−1.23	5.89	3.06E-26	3.50E-25
**uc001yxd.2**	8123	NA	PAR5	−2.31	4.63	9.06E-25	1.01E-23
**uc003pll.1**	23036	ENSG00000188994	ZNF292	−2.46	4.06	3.29E-20	3.34E-19
**uc009vvt.1**	643314	ENSG00000255103	KIAA0754	−0.97	5.72	1.10E-16	1.04E-15
**uc002uxh.1**	65072	ENSG00000226312	CFLAR-AS1	−1.78	5.89	8.70E-15	1.23E-13
**uc003lld.1**	100505658	ENSG00000246422	LOC100505658	−3.21	3.89	5.23E-13	4.51E-12
**uc010ggb.1**	26051	ENSG00000101445	PPP1R16B	−2.10	2.97	2.00E-11	1.64E-10
**uc002dkt.1**	641298	ENSG00000237296	SMG1P1	−1.79	3.52	1.09E-10	8.68E-10
**uc002diz.1**	100271836	ENSG00000180747	LOC100271836	−1.79	3.53	1.59E-10	1.25E-09
**uc002qqj.1**	84914	ENSG00000198466	ZNF587	−3.29	2.83	3.79E-10	2.97E-09
**uc002nvh.1**	2821	ENSG00000105220	GPI	−1.36	3.74	4.84E-09	3.63E-08
**uc003vwp.1**	50831	ENSG00000127362	TAS2R3	−2.95	2.33	8.98E-09	6.63E-08
**uc001gvt.1**	23046	ENSG00000116852	KIF21B	−0.97	4.29	9.90E-09	7.30E-08
**uc001xhy.1**	2877	ENSG00000176153	GPX2	−1.81	2.50	1.13E-08	8.24E-08
**uc002jtx.1**	10801	ENSG00000184640	41891	−0.99	4.21	6.09E-08	4.33E-07
**uc002syi.2**	23505	ENSG00000075568	TMEM131	−1.56	3.56	1.46E-07	1.01E-06
**uc002thr.1**	376940	ENSG00000188177	ZC3H6	−3.00	2.77	6.32E-07	4.23E-06
**uc002ixl.1**	388403	ENSG00000175155	YPEL2	−1.36	3.60	2.74E-06	1.76E-05
**uc002eoa.1**	677830	ENSG00000206952	SNORA50	−2.67	2.22	6.16E-06	3.88E-05
**uc003ezk.1**	4154	ENSG00000152601	MBNL1	−2.61	2.47	6.65E-06	4.17E-05
**uc001wtk.1**	253959	ENSG00000174373	RALGAPA1	−2.57	2.51	7.76E-06	4.82E-05
**uc003jkm.1**	25836	ENSG00000164190	NIPBL	−1.39	2.10	5.92E-06	4.94E-05
**uc010afy.1**	100131561	NA	FKSG29	−2.15	2.40	2.27E-05	1.62E-04
**uc002mto.2**	51710	ENSG00000197857	ZNF44	−1.24	3.64	1.12E-04	7.82E-04
**uc001dhy.1**	374986	ENSG00000180488	FAM73A	−1.04	3.27	1.20E-04	8.31E-04
**uc003kdz.2**	51426	ENSG00000122008	POLK	−2.04	1.85	1.20E-04	8.31E-04
**uc010des.1**	9931	ENSG00000198265	HELZ	−1.12	3.04	1.66E-04	9.22E-04
**uc010hnu.1**	6314	ENSG00000163635	ATXN7	−3.79	1.62	2.95E-04	1.59E-03
**uc009zgm.1**	5858	ENSG00000126838	PZP	−1.20	2.24	7.09E-04	3.67E-03
**uc003kxe.1**	3567	ENSG00000113525	IL5	−3.67	1.38	6.94E-04	4.30E-03

**Table 3:** Enrichment of GO molecular function terms in transcripts down-regulated as a result of globin depletion.

GOMFID	Pvalue	OddsRatio	ExpCount	Count	Size	Term
**GO:0046914**	1.32E-05	21.35748792	1.589655828	8	836	transition metal ion binding
**GO:0005344**	0.000116	187.2142857	0.01711352	2	9	oxygen transporter activity
**GO:0019825**	0.00067	68.81578947	0.039931546	2	21	oxygen binding
**GO:0046872**	0.000962	10.31220177	2.804715725	8	1475	metal ion binding
**GO:0008270**	0.001037	9.08266129	1.42612664	6	750	zinc ion binding
**GO:0043169**	0.001115	10.0253841	2.861760791	8	1505	cation binding
**GO:0030492**	0.001902	Inf	0.001901502	1	1	hemoglobin binding
**GO:0031720**	0.001902	Inf	0.001901502	1	1	haptoglobin binding
**GO:0020037**	0.005256	22.77192982	0.112188629	2	59	heme binding
**GO:0017123**	0.005695	291.5	0.005704507	1	3	Ral GTPase activator activity
**GO:0046906**	0.005791	21.62083333	0.117893136	2	62	tetrapyrrole binding
**GO:0005506**	0.006542	20.25390625	0.125499144	2	66	iron ion binding

## Discussion

Experimental globin depletion significantly reduced both the amount and quality of RNA isolated from PAXgene blood, but this did not have any measurable effect on our ability to construct a cDNA library from the extracted RNA. Indeed, there was no statistically significant difference in the total number of reads, total number of mapped reads or percentage of reads filtered out due to poor quality between GD samples and NGD samples. Conversely, both experimental pooling of the biological replicates and sequencing batch had a significant effect on the total number of reads and total number of mapped reads, and this was more pronounced in NGD samples. Experimental globin depletion removed a majority of globin transcripts (18% of reads mapping to globin transcripts in GD samples vs. 81% in NGD samples), but we note that the observed effectiveness was less than previously reported [7]. Globin depletion resulted in a significantly higher number of reliably detectable transcripts (FPKM≥1), in both technical and biological replicates. Technical reproducibility was high and was generally improved by globin depletion (rho_NGD lane replicates_ = 0.887, rho_GD lane replicates_ = 0.898; *p*<<0.01). In addition, while the correlation of pooled technical replicate pairs was worse than that of lane replicate pairs in NGD samples, it was not significantly worse in GD samples. Taken together, this suggests that globin depletion does not introduce significant bias to RNA-Seq data.

As expected, transcript FPKMs were generally higher following globin depletion, with 3500 additional transcripts reaching our chosen detection threshold (FPKM≥1) in GD samples compared to NGD samples in an exemplar biological replicate (mean number of additional transcripts reaching FPKM≥1 across samples = 5000). The majority of these newly detectable transcripts had low expression (FPKM≤10), though there were some notable outliers (FPKM≥10000). A number of non-globin transcripts were reproducibly depleted in GD biological replicates. Most of these transcripts were non-coding, and some exhibited highly variable expression across samples. Their reproducible depletion across all samples suggests that the globin depletion procedure itself is responsible, though why these particular transcripts were selectively depleted is not known. Unexplained, reproducible depletion of non-globin transcripts following globin depletion has been previously reported [7]. Assessing the effects of globin depletion on well annotated transcripts, mapping to unique gene symbols only, yielded more predictable results: over a thousand genes were consistently up-regulated as a result of globin depletion (FDR≤0.01), and only a handful of genes were consistently down-regulated across various experimental designs. Gene enrichment analysis results for these consistently down-regulated genes were predominantly driven by the presence of 2 hemoglobin genes (HBB and HBD; 9 of 12 significantly enriched GO molecular function terms at p≤0.05). The 3 remaining GO terms were driven by RALGAPA1 (Ral GTPase activator activity) and ZNF44, HELZ, ZC3H6, MBNL1, ZNF587, POLK, and ZNF292 (metal ion binding/zinc ion binding), respectively. Down regulation of zinc ion binding related genes as a result of experimental globin depletion has not been previously reported.

Overall, globin depletion appears to meaningfully improve the quality of peripheral whole blood RNA-Seq data, increasing the number of detectable transcripts (1.5–1000 FPKM range of expression values), while reducing the number of transcripts with marginal expression. Furthermore, it may improve our ability to detect true biological variation by reducing the technical variation inherent in peripheral whole blood RNA-Seq cDNA library preparation and sequencing. A key concern is the significant reduction in the amount of extracted RNA following experimental globin depletion. Biological samples are rarely easy to replace or replenish. Recommended library construction methods for RNA-Seq still require high amounts of starting RNA material and the loss following globin depletion may jeopardize the quality of the data from samples with only marginal amounts of available starting RNA material. Finally, while the cost of library construction in RNA-Seq experiments is considerable and relatively static, subsequent sequencing is rapidly becoming more affordable. With sufficient sequencing depth of coverage, it should be possible to replicate the effect of globin depletion without actually performing experimental depletion. Furthermore, sequential sequencing of a sample yields highly reproducible results. Therefore, in circumstances where RNA yields are necessarily low, it makes more sense to simply construct a library from all available RNA and sequence aliquots at the maximal depth of coverage one can afford. Additional depth will only become more affordable as time passes. This is particularly relevant to biobanking efforts. More generally, our investigation of technical and biological variation with and without globin depletion finds that high-throughput sequencing by RNA-Seq is highly reproducible within a large dynamic range of detection and provides an accurate estimation of RNA concentration in peripheral whole blood. High-throughput sequencing is thus a promising technology for whole blood transcriptomics and biomarker discovery.

## Supporting Information

Figure S1 **Library size and number of mapped reads for NGD and GD samples.** Total number of reads (right axis) and robustly detectable transcripts (FPKM≥1; left axis) are plotted for all samples, for both sequencing lanes separately.
(TIFF)

Figure S2 **ERCC spike-in control transcripts correlation in NGD and GD samples.** Linear response of ERCC spike-in transcripts for 2 pooled technical replicates, sequenced at different times, on separate lanes.
(TIFF)

Table S1 **Effect of Globin Depletion on RNA Quality and cDNA Library Size in Technical Replicates (detail).**
(XLSX)

Table S2 **Effect of Globin Depletion on RNA Quality and cDNA Library Size in Biological Replicates (detail).**
(XLSX)
