# Supplementary figures and images for: Variation in RNA-Seq Transcriptome Profiles of Peripheral Whole Blood from Healthy Individuals with and without Globin Depletion
Source: PLoS One. 2014 Mar 7;9(3):e91041. doi: 10.1371/journal.pone.0091041 (PMC3946641; doi:10.1371/journal.pone.0091041)

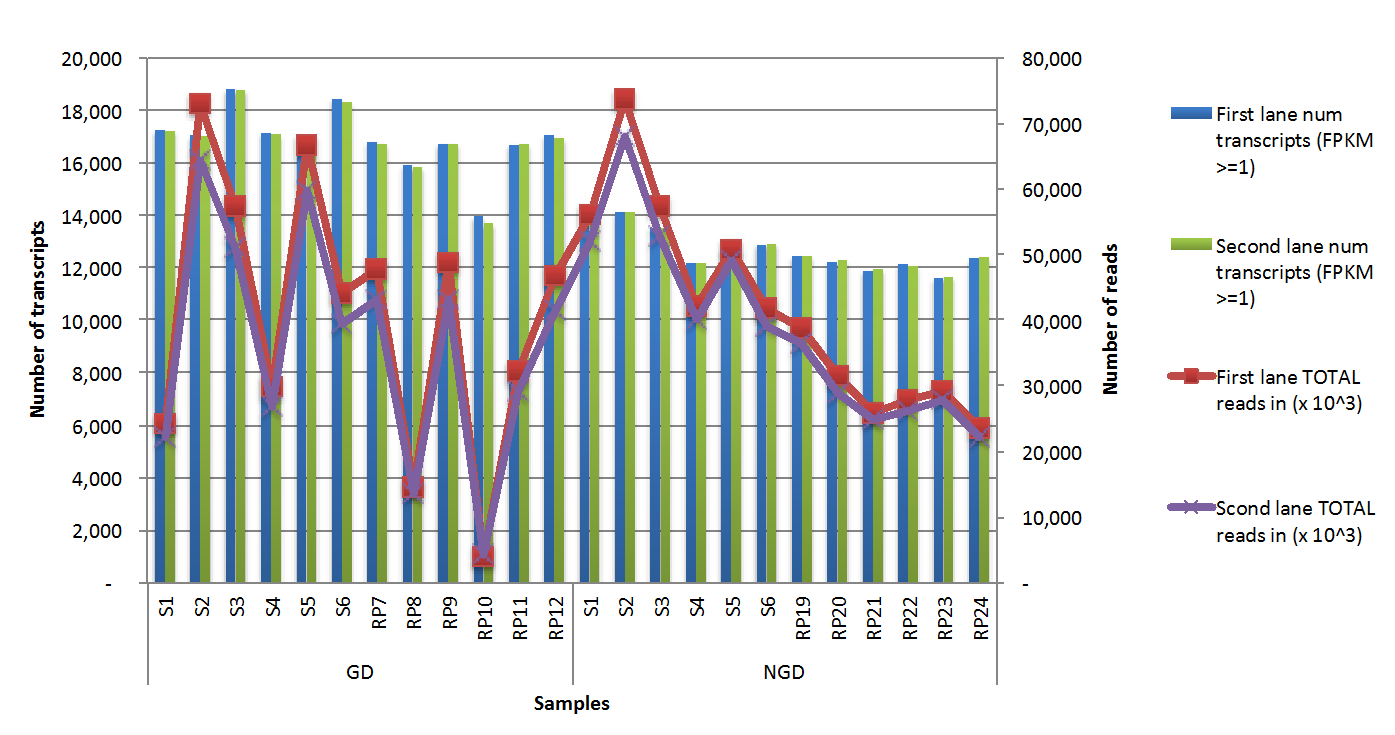

Supplement: Figure S1 — Library size and number of mapped reads for NGD and GD samples. Total number of reads (right axis) and robustly detectable transcripts (FPKM≥1; left axis) are plotted for all samples, for both sequencing lanes separately. (TIFF) [file pone.0091041.s001.tiff]

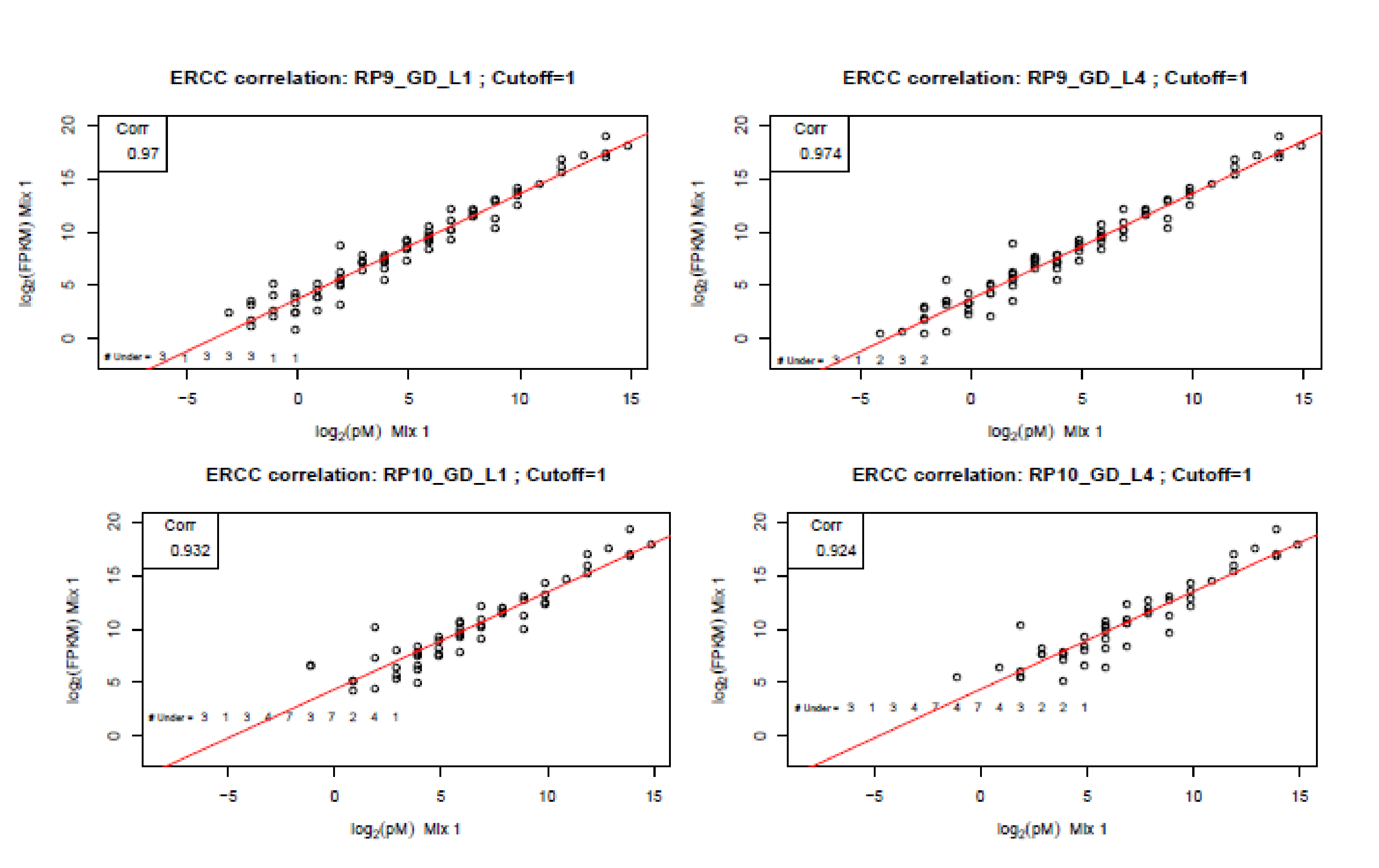

Supplement: Figure S2 — ERCC spike-in control transcripts correlation in NGD and GD samples. Linear response of ERCC spike-in transcripts for 2 pooled technical replicates, sequenced at different times, on separate lanes. (TIFF) [file pone.0091041.s002.tiff]
